# Effects of Late-Life Caloric Restriction on Age-Related Alterations in the Rat Cortex and Hippocampus

**DOI:** 10.3390/nu13010232

**Published:** 2021-01-15

**Authors:** Claudia Tonini, Marco Segatto, Francesca Martino, Luisa Cigliano, Martina Nazzaro, Laura Barberio, Maurizio Mandalà, Valentina Pallottini

**Affiliations:** 1 Department of Science, University Roma Tre, Viale Marconi 446, 00146 Rome, Italy; claudia.tonini@uniroma3.it (C.T.); francymartino@alice.it (F.M.); 2 Department of Biosciences and Territory, University of Molise, Contrada Fonte Lappone, 86090 Pesche, Italy; marco.segatto@unimol.it; 3 Department of Biology, University of Naples Federico II, Complesso Universitario Monte Sant’Angelo, Via Cinthia—Edificio 7, 80126 Naples, Italy; luisa.cigliano@unina.it (L.C.); martina.nazzaro@unina.it (M.N.); 4 Department of Biology, Ecology and Earth Science, University of Calabria, Arcavacata di Rende, 87036 Cosenza, Italy; laura.barberio90@gmail.com (L.B.); m.mandala@unical.it (M.M.); 5 Neuroendocrinology Metabolism and Neuropharmacology Unit, IRCSS Fondazione Santa Lucia, Via del Fosso Fiorano 64, 00143 Rome, Italy

**Keywords:** aging, caloric restriction, cholesterol, cortex, hippocampus, rats

## Abstract

Background: A major problem of aging is the disruption of metabolic homeostasis. This is particularly relevant in the brain where it provokes neurodegeneration. Caloric restriction is a physiologic intervention known to delay the deleterious consequences of aging in several species ranging from yeast to mammals. To date, most studies on experimental models have started this dietary intervention from weaning, which is very difficult to be translated to human beings. Here, we study the effects of a more realistic dietary regimen in rats, starting at an advanced age and lasting for six months. Methods: we analyzed in the cortex and hippocampus, the proteins involved in the energetic balance of the cells, cholesterol metabolism, oxidative stress response, inflammation, synaptic impairment, and brain trophism. Results: our results suggest that caloric restriction in late life can revert only some age-related changes studied here.

## 1. Introduction

Aging entails a progressive decline in physiological functions of the body and increases the probability of getting sick and of dying [1]. A key change is the loss of homeostasis that modifies the molecular composition of tissues and reduces the ability to adapt to environmental changes, which in turn increases the susceptibility and vulnerability to diseases [2]. Research within the last decades identified several mechanisms at the base of aging including genomic instability, loss of proteostasis, deregulated nutrient sensing, mitochondrial dysfunction, and alterations of epigenetic and intracellular signaling pathways. Moreover, increased inflammation, decreased stem cell amount, and accumulation of senescent cells have been postulated [3]. Cells in the brain are particularly sensitive to aging. As an example, aging decreases the energy status of cells [4] and disrupts lipid homeostasis, namely cholesterol metabolism [5]. Other alterations in the brain include increased oxidative stress [6] and inflammation [7], synaptic impairment [8], and decreased brain trophism [9].

A key challenge for biomedical research is to find ways to reduce or prevent changes due to aging. Nearly 90 years ago, it was shown that a reduction of caloric intake in laboratory rats extends their life span [10]. Subsequent studies confirmed this effect in species ranging from yeast to mammals and showed that it was due to a reversal of age-related alterations [11,12,13]. In the rodent brain, CR ameliorates learning, memory, and behavioral performances, and it augments synaptic plasticity and neurogenesis [4]. However, most of these results were obtained in mammals subjected to CR starting from weaning [4,14]. Evidently, this paradigm cannot be easily translated to human beings, as it would involve life-long changes in dietary and cultural habits. Therefore, a key challenge is to determine the effects of CR starting later in life [15]. To date, information regarding the effects of this CR regimen especially on the central nervous system (CNS) is very limited. Our study aimed to close this gap. To this end, we studied the effects of CR starting at an advanced age and compared age- and CR-induced changes using three-month-old rats as a control group. We focused on proteins involved in key processes modulated by aging, namely the energetic status of the cells, cholesterol metabolism, oxidative stress response, inflammation, synaptic impairment, and brain trophism.

## 2. Materials and Methods

### 2.1. Animals

Experiments were performed on 13-week-old adult (3M, *n* = 5) and 98-week-old (24M, *n* = 10) male Sprague–Dawley rats. Animals were housed in light- (12:12 h light-dark cycle) and temperature (22 °C)-controlled rooms, with free access to food and water. Animals were fed with a standard laboratory chow (ssniff diet V1535, metabolizable energy 3.057 Kcal/kg) until they were euthanized with isoflurane (4%) followed by cervical transection. Brains were removed immediately and stored at −80 °C until further use. All experiments were carried out in accordance with the European Guidelines for the care and use of laboratory animals (Directive 26/2014/EU), and they were approved by the local ethical committee of the University of Calabria and by the Italian Ministry of Health (license n.295/2016-PR).

### 2.2. Caloric Restriction

At 72 weeks of age, the rats were divided into two subgroups: (1) Control rats, who had continued access to ad libitum diet (normal diet, ND *n* = 5), and (2) Caloric Restricted rats (CR, *n* = 5), who received the same chow but 40% less than the intake in age-matched ND rats. CR was imposed for a total period of 6 months. Animals were sacrificed at 24 Months of age (24M ND and 24M CR). Water and food intake were recorded every other day, while body mass was recorded monthly.

### 2.3. Cortex Cholesterol Content

The cholesterol amount in tissue samples was measured using the Cholesterol Quantitation Kit-MAK043 following the manufacturer’s instructions (Sigma Aldrich, Milan, Italy).

### 2.4. Total lysate and Membrane Preparation for Western Blot Analysis

Hippocampi and cortices were dissected and lysed in 1:5 *w*/*v* homogenization buffer (Sucrose 0.1 M, KCl 0.05 M, KH_2_PO_4_ 0.04 M, EDTA 0.04 M, pH 7.4, with 1:1000 protease inhibitor cocktail and 1:400 phosphatase inhibitor cocktail, Sigma-Aldrich) on ice by sonication for 30 s (VCX 130 PB, Sonics, Newtown, 06470 CT), and centrifuged at 10,000 rpm for 10 min, at 4 °C. To isolate membrane fractions, total lysate was centrifuged at 14,000 rpm for 1 h at 4 °C and the pellet was solubilized in homogenization buffer by sonication. Protein concentration was assessed by the method of Lowry [16]. Aliquots of homogenate samples were diluted with Laemmli buffer, boiled for 5 min, and subjected to sodium dodecyl sulfate polyacrylamide gel electrophoresis (SDS-PAGE) for subsequent Western blot analysis.

### 2.5. Immunoblotting

Proteins (15 μg) from total lysates were separated by SDS-PAGE at 50 mA (constant current) first, and then at 120 V for 120 min. Subsequently, proteins were transferred to nitrocellulose membranes using the Trans-Blot Turbo Transfer System (Bio-Rad Laboratories). The nitrocellulose membranes were incubated at room temperature with 5% fat-free milk in Tris-buffered saline (NaCl 0.138 M, KCl 0.027 M, Tris-HCl 0.025 M, and 0.05% Tween-20, pH 6.8), and then overnight, at 4 °C, with primary antibody, followed by 1 h of incubation with secondary peroxidase-conjugated antibody produced in mouse or in rabbit (1:10,000; Biorad). Immunoreactivity was detected using the clarity ECL Western blotting system (Bio-Rad Laboratories) and a ChemiDoc MP system (Bio-Rad Laboratories). The following primary antibodies were used: HMGCR (Abcam, ab242315, dilution 1:1000), P-HMGCR Ser872 (Merck Millipore, #09-356, dilution 1:1000), P-AMPK Thr172 (Sigma-Aldrich, #15-115, dilution 1:1000), RhoA (Santa Cruz Biotechnology, Santa Cruz, CA, sc-418, dilution 1:500), Ras (Santa Cruz Biotechnology, sc-53959, dilution 1:500), LDLR (Abcam, ab30532, dilution 1:1000), nSREBP2 (Abcam, ab30682, dilution 1:1000), BDNF (Santa Cruz Biotechnology, sc-546, dilution 1:1000), Iba1 (Santa Cruz Biotechnology, sc-32725, dilution 1:1000), ABCA1 (Santa Cruz Biotechnology, sc-58219, dilution 1:1000), CYP46A1 (Santa Cruz Biotechnology, sc-1361148, dilution 1:1000), Synaptotagmin (Cell Signaling, #14558, dilution 1:1000), APOE (Merck Millipore, ab947, dilution 1:500), Nox2 (Santa Cruz Biotechnology, sc-130543, dilution 1:1000), and p47phox (Santa Cruz Biotechnology, sc-17844, dilution 1:1000). As loading controls, immunoblots were reacted with antibodies against tubulin, vinculin, or actin (1:10,000; Sigma Aldrich). To avoid confounding band signals during the immunoreactivity detection, we used different housekeeping proteins in dependence of the molecular weight of the protein of interest detected on the same nitrocellulose membrane. Western blot images were analyzed by ImageJ (National Institutes of Health, Bethesda, MD, USA) software for Windows. Intensities of proteins of interest were normalized to intensities of respective housekeeping proteins.

### 2.6. Statistical Analyses

Data are expressed as mean ± SD for all experiments. The experiments were performed in duplicate (technical replicates) and at least four animals per experimental group were used (biological replicates). Statistically significant differences were tested by one-way analysis of variance (ANOVA) followed by Tukey’s post hoc test. Statistical analyses and graph editing were performed using GRAPHPAD Prism6 (GraphPad, La Jolla, CA, USA) for Windows.

## 3. Results

We aimed to test whether CR starting at an advanced age can reverse aging-induced changes in key proteins in brain health. To this end, the hippocampus and the cortex of 24-month-old rats subjected to CR for 6 months were used and compared to those of 3- and 24-months-old rats fed with normal diet (ND).

Rats subjected to between 18 and 24 months of CR showed significantly reduced body weights compared to age-matched littermates, indicating that CR was effective. However, the weight of CR rats was still increased compared to the weight of animals at3Months of age (Table 1).

**Figure 1:**
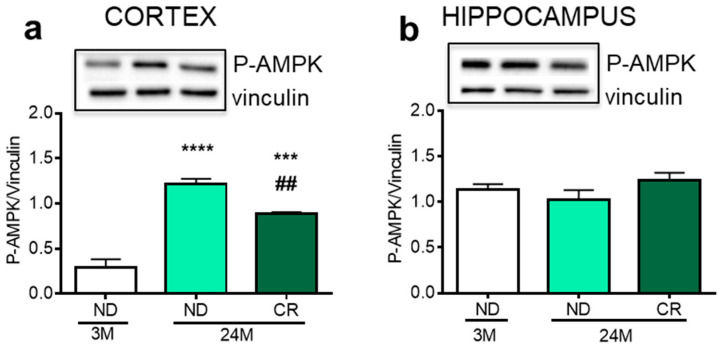
Impact of aging and reduction of caloric intake (CR) on phosphorylated AMPK levels in the rat cortex and hippocampus. Representative Western blots and densitometric analyses of phosphorylated AMPK (P-AMPK) in cortex (**a**) and hippocampus (**b**), using vinculin as housekeeping protein for loading control. 3M (3-month-old rats); 24M (24-month-old rats); ND (normal diet); CR (caloric intake reduced by 40% for the last six months of life). Statistical analysis was performed by using one-way ANOVA followed by Tukey’s post hoc test. **** *p* < 0.0001, *** *p* < 0.001 vs. ND 3M; ^##^ *p* < 0.01 vs. ND 24M.

First, we analyzed the impact of CR on the level of phosphorylated AMP-activated kinase (AMPK), a well-known sensor of energy whose phosphorylation state depends on the AMP/ATP ratio [17]. In the cortex, the level of phosphorylated—and thus active—AMPK increased during aging (*p* < 0.0001, Df 12, q = 14.53; *p* < 0.01; Df = 12, q = 9.324), and this change was partially reverted by CR (*p* < 0.0082; Df = 12; q = 5.201 vs. 24M ND) (Figure 1a). On the other hand, phosphorylated AMPK (P-AMPK) levels in the hippocampus were unaffected by age or CR (Figure 1b).

**Figure 2:**
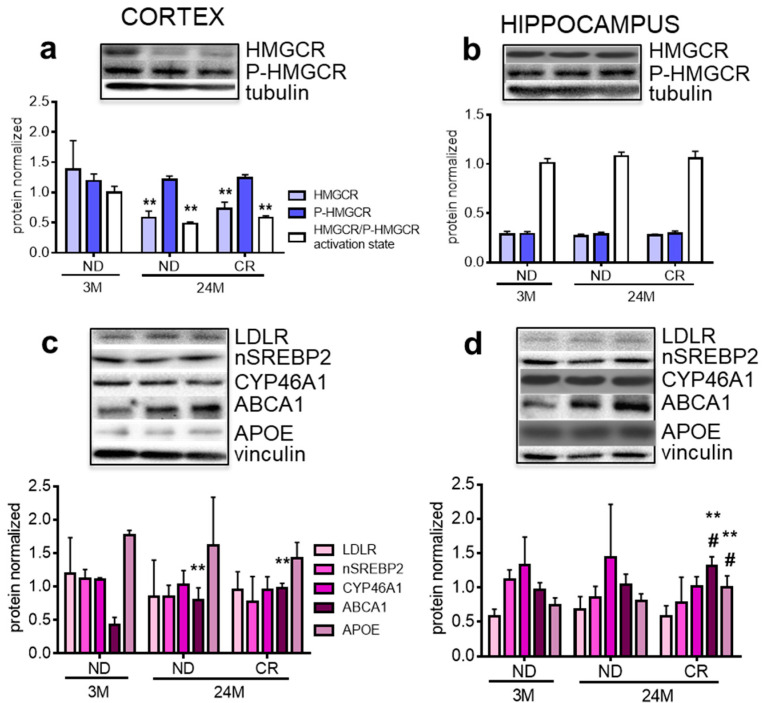
Impact of age and CR on proteins handling cholesterol metabolism in rat cortex and hippocampus. Panels (**a**,**b**) show representative Western blots and densitometric analyses of HMGCR (total and phosphorylated protein) in the cortex and hippocampus, respectively. Panel **c** and **d** show representative Western blots and densitometric analysis of other proteins involved in cholesterol homeostasis in the cortex and hippocampus, respectively. 3M (3-month-old rats); 24M (24-month-old rats); ND (normal diet); CR (caloric intake reduced by 40% for the last 6 months of life). Protein levels were normalized to tubulin (**a**,**b**) and to vinculin (**c**,**d**). Statistical analysis was performed by using one-way ANOVA followed by Tukey’s post hoc test. ** *p* < 0.01 vs. ND3M ND; ^#^ *p* < 0.05 vs. 24M ND.

Next, we analyzed proteins of cholesterol metabolism which can be affected by aging [18]. In particular, age-dependent dysregulation of 3-hydroxy 3-methylglutaryl Coenzyme A reductase (HMGCR), the key and rate-limiting enzyme of cholesterol biosynthesis, has been demonstrated in rats [19,20]. The activity of this enzyme is inhibited by phosphorylation at Ser872 [21]. Therefore, we determined the ratio between total HMGCR and phosphorylated HMGCR (P-HMGCR), which mirrors the active fraction of the enzyme. The levels of total HMGCR and its active fraction declined with age in both 24M ND rats (*p* < 0.01, Df = 9; q = 4.547 vs. 3M ND) and 24M CR rats (*p* < 0.01; Df = 9; q = 4.198 vs. 3M ND), in the cortex but not in the hippocampus. Thus, no changes were induced by CR (Figure 2a). To obtain a comprehensive view, we tested other proteins involved in cholesterol metabolism: (1) low density lipoprotein receptor (LDLR), which is involved in lipoprotein containing cholesterol internalization in the cells [22]; (2) the transcriptional active fragment of sterol regulatory element binding protein 2 (nSREBP2), which is committed to the transcription of genes involved in cholesterol homeostasis [23]; (3) cholesterol 24-hydroxylase (CYP46A1), the rate limiting enzyme for cholesterol degradation [24]; (4) ATP binding cassette A1 (ABCA1), which controls the efflux of cholesterol from cells [25]; and (5) apolipoprotein E (APOE), which is the major cholesterol carrier in the brain [26,27].

In the cortex, we observed an age-dependent increase of ABCA1 both in 24M ND (*p* < 0.01; Df = 9; q = 6.093) and in 24M CR (*p* < 0.01; Df = 9; q = 6.022), as compared to 3M ND, which was not affected by CR (Figure 2c). In the hippocampus, CR increased the levels of ABCA1 (*p* < 0.01, Df = 9; q = 6.022 vs. 3M ND; *p* < 0.05; Df = 9; q = 4.714 vs. 24M ND) and APOE (*p* < 0.01, Df = 9; q = 6.683 vs. 3M ND; *p* < 0.05; Df = 9; q = 4.333 vs. 24M ND). None of the other proteins tested was affected by age or CR.

Since the activation state of HMGCR was reduced in cortex, we tested whether this alteration affected end-products of the biosynthetic pathway in this specific area. Our results show that the cholesterol content was not affected by age or CR (Table 2).

**Figure 3:**
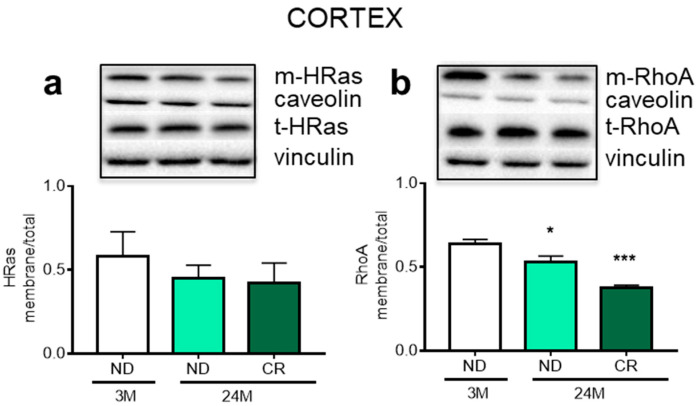
Impact of age and CR on membrane-bound and total levels of HRas and RhoA in rat cortex. Representative Western blots and densitometric analysis of membrane-bound (m-) and total content (t-) of HRas (**a**) and RhoA (**b**) in rat cortex. Vinculin and caveolin served as housekeeping proteins to normalize protein loading. 3M (3-month-old rats); 24M (24-month-old rats); ND (normal diet); CR (caloric intake reduced by 40% for the last 6 months of life). Statistical analysis was performed by using one-way ANOVA followed by Tukey’s post hoc test. * = *p* < 0.05, *** = *p* < 0.001, vs. 3M ND.

The HMGCR-dependent mevalonate pathway produces prenyls, such as farnesol (F) and geranylgeraniol (GG), that are required for the post-translational modification of signaling proteins. Notably, the covalent attachment of GG and F assures the membrane anchoring and thus the activation of the small GTPases RhoA and HRas, respectively [28]. The level of membrane-bound RhoA was decreased by age (*p* < 0.05; Df = 12; q = 5.341 vs. 3M ND). This change was not reversed by CR (*p* < 0.001; Df = 12; q = 8.811 vs. ND) (Figure 3a), in agreement with the HMGCR reduced activation observed in the cortex. The levels of membrane-bound and total HRas were not affected by aging or CR (Figure 3b).

**Figure 4:**
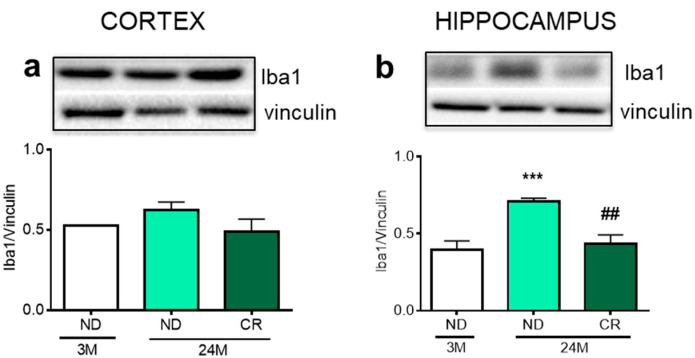
Impact of age and CR on the microglia marker Iba1 in rat cortex and hippocampus. Representative Western blots and densitometric analysis of Iba1 levels in the cortex (**a**) and hippocampus (**b**) normalized to vinculin. 3M (3-month-old rats); 24M (24-month-old rats); ND (normal diet); CR (caloric intake reduced by 40% for the last 6 months of life). Statistical analysis was performed by using one-way ANOVA followed by Tukey’s post hoc test. *** *p* < 0.001 vs. 3M ND; ^##^ *p* < 0.01 vs. 24M ND.

Next, we evaluated the expression of ionized calcium-binding adapter 1 (Iba 1), a marker of microglial cells [27]. Our analyses revealed no age-dependent alteration of Iba1 in the cortex (Figure 4a). In the hippocampal formation, the protein increased with age (*p* < 0.05; Df 12; q = 4.045 vs. 3M ND) and this effect was reverted by CR (*p* < 0.001; Df = 12; q = 8.118 vs. 24M ND) (Figure 4b).

**Figure 5:**
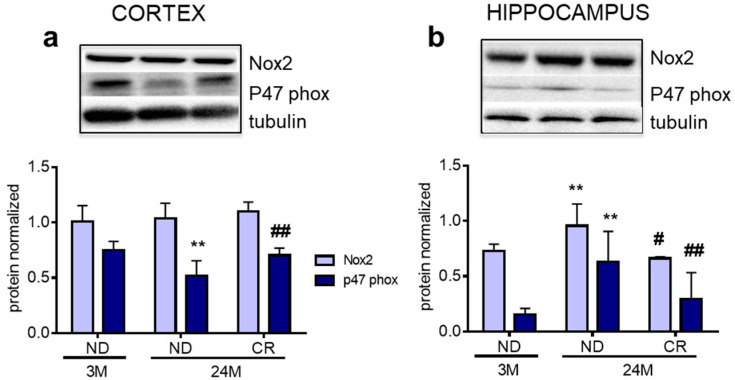
Impact of age and CR on Nox2 and p47phox proteins in rat cortex and hippocampus. Representative Western blots and densitometric analyses of Nox2 and p47phox levels in the cortex (**a**) and hippocampus (**b**). Tubulin served as housekeeping protein to normalize protein loading. 3M (3-month-old rats); 24M (24-month-old rats); ND (normal diet); CR (caloric intake reduced by 40% for the last 6 months of life). Statistical analysis was performed by using one-way ANOVA followed by Tukey’s post hoc test. ** *p* < 0.01 vs. 3M ND, ^#^ *p* < 0.05; ^##^ *p* < 0.01 vs. 24M ND.

Next, we explored effects of age and CR on oxidative stress using components of the the NADPH oxidase complex, the major endogenous sources of reactive oxygen species (ROS) during aging [29]. The transmembrane protein Nox2 was not affected by age or CR in the cortex (Figure 5a), but it showed an age-dependent increase in the hippocampus, which was reverted by CR (*p* < 0.01; Df = 9; q = 5.003 vs. 3M ND. *p* < 0.05; Df = 9; q = 4.343 vs. 24M ND) (Figure 5b). In contrast, the cytosolic p47phox [30] showed an age-dependent increase in the cortex (*p* < 0.01; Df = 9; q = 5.417 vs. 3M ND), while it was decreased in the hippocampus (*p* < 0.01; Df = 9; q = 4.903 vs. 3M ND). Remarkably, both changes were reverted by CR (Nox2: *p* < 0.01; Df = 9; q = 4.339 vs. 24M ND; p47phox *p* < 0.01; Df = 9; q = 5.893 vs. 24M ND).

**Figure 6:**
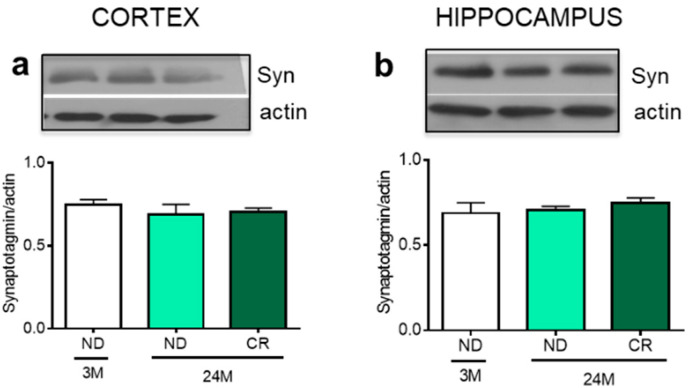
No effect of age and CR on Synaptotagmin in rat cortex and hippocampus. Representative Western blots and densitometric analyses of synaptotagmin in the cortex (**a**) and hippocampus (**b**) normalized to actin levels as loading control. 3M (3-month-old rats); 24M (24-month-old rats); ND (normal diet); CR (caloric intake reduced by 40% for the last 6 months of life).

We further studied the level of synaptotagmin, a marker of synapses [31]. The result shown in Figure 6 demonstrated that neither aging nor CR modify the level of this protein in the cortex or hippocampus.

**Figure 7:**
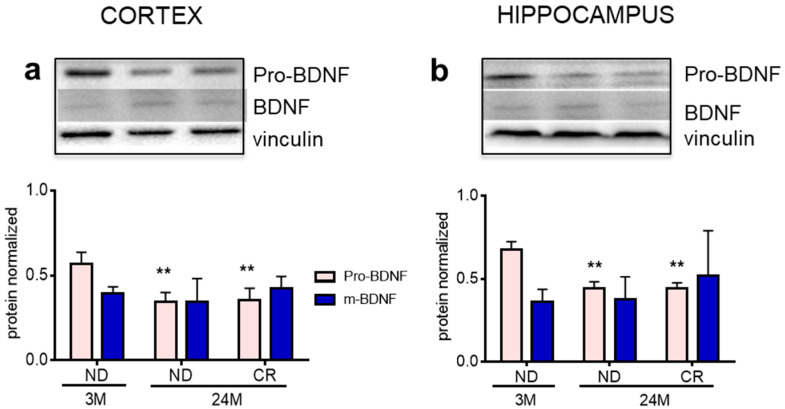
Impact of age and CR on levels of brain-derived neurotrophic factor (BDNF) and pro-BDNF in rat cortex and hippocampus. Representative Western blots and densitometric analyses of pro-BDNF and BDNF in the cortex (**a**) and hippocampus (**b**) normalized to vinculin. 3M (3-month-old rats); 24M (24-month-old rats); ND (normal diet); CR (caloric intake reduced by 40% for the last 6 months of life). Statistical analysis was performed by using one-way ANOVA followed by Tukey’s post hoc test. ** = *p* < 0.01 vs. ND3M.

Lastly, we measured the level of brain-derived neurotrophic factor (BDNF), a neurotrophin that controls neuronal survival, differentiation, and function during brain development and in adulthood [32]. The levels of the precursor pro-BDNF decreased with age in both brain regions (cortex: *p* < 0.01; Df = 9; q = 5.621 vs. 3M ND; hippocampus: *p* < 0.01; Df = 9; q = 5.234 vs. 3M ND), without reversal of these changes by CR (cortex: *p* < 0.01; Df = 9; q = 5.354 vs. 3M ND; hippocampus: *p* < 0.01; Df = 9; q = 5.841 vs. 3M ND) (Figure 7a,b).

## 4. Discussion

The impact of aging varies between individuals and the biological mechanisms are only partially understood [3]. The CNS is the organ most affected by aging, showing highly heterogeneous changes. Aging alters the structure, metabolism, and physiology of the brain, and drives cognitive and neurologic dysfunctions [33].

On the other hand, it is known that the progress of aging can be modulated, and that diet is one of the factors able to interfere, positively or negatively, with this physiological process. Several studies showed that a reduced caloric intake can delay aging and maintain a healthy status in numerous animal species [34]. In mammals, it has been extensively demonstrated that caloric restriction, starting from weaning, counteracts age-dependent molecular and cellular alterations, and preserves synaptic plasticity and neurogenesis, thus improving learning and memory [35]. However, a dietary intervention starting from weaning is not applicable to humans. To study a more realistic scenario, we evaluated the effects of CR in rats starting at 18 months of age, which can be compared to a 60-year-old human being, up to and lasting until 24 months of age, which corresponds to almost 80 years of age in humans.

A key feature of neurons is their high metabolic rate [36], which is required to ensure electrical activity in distant compartments. AMPK is the principal component for energy homeostasis in eukaryotic cells [37] and thus this enzyme has a critical role in neuronal survival. Our finding that AMPK phosphorylation is increased by aging and mitigated by CR in brain cortex suggests an age-dependent disruption of energy balance which is partially restored by CR. This data does not match with some studies, showing that a long-term CR induces an increased AMPK activation. In fact, it has been demonstrated that AMPK activation inhibits the mammalian target of rapamycin (mTOR) cascade and acts as a molecular transducer of beneficial starvation signals in both lower and higher eukaryotes [38,39]. Moreover resveratrol, a widely recognized CR mimetic, exerts its effects in a fashion that involves AMPK activation [40]. Thus, our results deserve further investigations to understand the reason why a short-term CR determines this specific alteration in the cortex, and to explain the apparent resistance to energy changes of the hippocampus both during aging and CR administration.

Cholesterol homeostasis is essential for the CNS and alterations have been associated with age-related neurodegenerative diseases including Parkinson’s, Alzheimer’s, and Huntington’s [41]. Furthermore, we previously demonstrated that this metabolic pathway was altered during aging, but the study was carried out on the whole rat brain [19]. Our present data show clear evidence for region-specific impact of aging and CR on cholesterol metabolism. The cortex showed an age-dependent reduction of HMGCR activation and increased expression of ABCA1, but no impact of CR. On the contrary, CR increased the level of ABCA1 in the hippocampus, confirming that different brain areas differently respond to the same stimulus [20]. A limitation of this study stems from the fact that the cell types causing these changes cannot be identified. However, we can speculate that the increased ABCA1 levels observed in the cortex could represent a compensatory effect due to the lower HMGCR activation which, however, is not accompanied by a concurrent reduction in cholesterol content. This apparent discrepancy is explained by the fact that the amount of brain cholesterol is very stable, since its turnover represents the 0.4% per day in mouse brains and even 0.03% per day in human brains [42]. In line with Perovic and collaborators [43] we did not observe age-dependent changes of APOE protein levels. However, in contrast to their findings, we found a CR-induced increase of APOE in the hippocampus, which was previously shown to be an area particularly sensitive to diet-induced changes in the cholesterol protein network [27]. The increase of this protein, as well as ABCA1, may ameliorate cholesterol trafficking between glial cells and neurons, avoiding brain cholesterol-accumulation or altered signaling associated with aging. The age-dependent reduction of membrane-bound RhoA, which was not restored by CR, strongly corroborates the reduced HMGCR activation state. Given that RhoA activation or inactivation is involved in neurite retraction or outgrowth, respectively [44,45], our observations suggest alterations in neurite formation or maintenance.

Aging is accompanied by increased oxidative stress and production of ROS [46] to which brain regions show different degrees of vulnerability [47]. Interestingly, we observed that region-specific changes of p47phox and Nox2 levels were completely restored by CR. In addition, CR was also able to restore the levels of Iba1 in the hippocampus, indicating that six months of CR at advanced age are sufficient to restore the age-dependent changes in these components.

The precursor and mature form of neurotrophin BDNF are key signals for brain function as they control numerous signaling pathways through distinct receptors, TrkB and p75NTR, respectively. In fact, while the mature form promotes long-term potentiation, pro-BDNF facilitates long-term depression. Thus, a bidirectional control of synaptic plasticity is guaranteed by the expression pattern of the receptors and by a balanced proportion of the two BDNF species [32]. As a consequence, an imbalance of the two forms could induce consistent functional modifications. Our results show that aging induces a decrease of pro-BDNF in both cortex and hippocampus, leading to an altered ratio of the two neurotrophin forms, and that these changes are not reversed by CR.

Altogether, our results show that a CR regimen that starts at advanced age and lasts for 6 months, representing a more realistic regimen than life-long fasting, reverts some aging defects with clear region-specific effects. This includes restoration of energy metabolism and abolition of microglia activation in the hippocampus, as well as reduction in some oxidative stress parameters. This regional difference in vulnerability probably relies on the different cytoarchitectures and functions of the two regions. As shown previously, brain areas respond differently to the same physiologic stimulus [20,27]. A weakness of our study is that we cannot trace back the observed changes to the cells of origin. Moreover, in this work, we did not take into consideration the putative sex-dependent differences; in fact, most of the analyzed physiologic parameters are regulated in a sex-dependent manner [2,48,49,50,51]. So, these aspects need to be further investigated.

## Figures and Tables

**Table 1:** Animal weights at sacrifice.

	3M ND	24M ND	24M CR
**Animal weight g**	250 ± 21	444 ± 42 ***	370 ± 23 ***^,##^

*** *p* < 0.001 vs. 3M ND; ^##^ *p* < 0.01 vs. 24M ND as from one-way ANOVA followed by Tukey’s post hoc test. 3M (3-month-old rats); 24M (24-month-old rats); ND (normal diet); CR (Caloric Restricted (caloric intake reduced by 40% for the last six months of life)).

**Table 2:** Cholesterol content in the Cortex.

	3M ND	24M ND	24M CR
**Cholesterol µg/mg tissue**	4.77± 0.05	5.43 ± 0.8	4.65 ± 0.32

3 M (3-month-old rats); 24M (24-month-old rats); ND (normal diet); CR (caloric intake reduced by 40% for the last 6 months of life).

## Data Availability

The data presented in this study are available on request from the corresponding author.
